# Basic Sites on Alumina with Preadsorbed Ethanol and Ammonia—An IR Study

**DOI:** 10.3390/molecules29081726

**Published:** 2024-04-11

**Authors:** Jerzy Podobiński, Jerzy Datka

**Affiliations:** Jerzy Haber Institute of Catalysis and Surface Chemistry, Polish Academy of Sciences, Niezapominajek 8, 30-239 Krakow, Poland; jerzy.podobinski@ikifp.edu.pl

**Keywords:** alumina, basic sites, CO_2_ adsorption, ethoxy groups, ammonia adsorption

## Abstract

The adsorption of ethanol and ammonia changes the basic properties of alumina, and new basic sites are created. Ethanol reacts with surface Al-OH groups, forming ethoxy group Al-O-C_2_H_5_. The substitution of Al-OH by Al-O-C_2_H_5_ increases the negative charge of neighbouring oxygen atoms, and they became sufficiently basic to react with adsorbed CO_2_ forming carbonate species CO_3_^2−^. These carbonates were found to be monodentate and bidentate species. Preadsorption of ammonia also increases the basicity of alumina, but the mechanism is different than for ethanol adsorption. Adsorbed ammonia interacts with surface Lewis acid sites being three-coordinated aluminium atoms. This interaction is accompanied by an electron transfer from ammonia molecules to surface sites, and increases the basicity of the neighbouring oxygens, which can react with the absorbed CO_2_. The carbonate species formed are polydentate ones.

## 1. Introduction

Basic catalysts play an important role in the chemical industry. They catalyse various reactions of organic molecules, such as hydrogenation, double-bond isomerization, dehydrocylcodimerization, amination, aldol condensation, Michael addition, nitroaldol reaction, Tishchenko reaction, conjugate addition of alcohol, cyanoetylation, and others [1,2,3].

Therefore, the recognition of basic properties of catalysts (or, more generally, of solids) is important for science and technology. In practically all of the studies, CO_2_ has been used as probe molecule [4,5,6,7,8,9,10,11,12,13,14,15,16,17,18,19].

The oxygen atoms and hydroxyl groups on metal oxides have a negative charge. Generally, the less electronegative the metal is, the more negative both the oxygens and surface hydroxyls are. These sites are potential basic sites. However, the local environment of surface sites may cause bigger polarization of the M-O and M-OH bond; therefore, these sites are more basic than other ones, and these O^2−^ or OH^−^ may react with adsorbed CO_2_ molecules, forming carbonate species (CO_3_^2−^) or bicarbonate species (HCO_3_^−^). Our earlier IR [18] study evidenced that, for alumina, only ca. 5–10% of surface hydroxyls was able to react with CO_2_, forming bicarbonate species, and surface oxygens practically did not react with CO_2_. On the other hand, ca. 30% of hydroxyls situated on zirconia surface reacted with CO_2_, forming bicarbonate, and some surface oxygens also reacted with CO_2_, forming carbonates. Bigger reactivity of surface sites on zirconia with CO_2_, if comparing with alumina, may be related to lower electronegativity of Zr if comparing with Al (1.33 and 1.61, respectively). However, for both alumina and zirconia, only a relatively small fraction of surface sites is sufficiently basic to react with CO_2_ forming bicarbonate and carbonate species. The surface hydroxyls reacting with CO_2_ will be denoted as OH^−^, and surface oxygens reacting with CO_2_ will be denoted as O^2−^.

The adsorption studies and TPD experiments provide information on the total concentration of all the basic sites, O^2−^ plus OH^−^, without distinguishing between them. On the other hand, IR spectroscopic studies of CO_2_ adsorption can discriminate between O^2−^ and OH^−^. O^2−^ reacting with CO_2_ produces carbonate species (CO_3_^2−^), whereas OH^−^ forms bicarbonates (HCO_3_^−^). Carbonate and bicarbonate species show different IR spectra [10,13,15,16,17]. Both carbonates and bicarbonates may be bonded to surface sites in different ways. They may be monodentate, bidentate, or polydentate. Therefore, a careful analysis of the IR spectrum of adsorbed CO_2_ informs not only which kind of basis sites (O^2−^ or OH^−^) are present on the surface, it also provides additional information how the (bi)carbonate species are bonded to adsorption sites. However, the IR results concerning CO_2_ adsorption obtained by the above-mentioned authors have only qualitative aspects. Recently, we elaborated [18] a new method of quantitative IR studies of concentrations of both O^2−^ and OH^−^. The extinction coefficients of diagnostic IR bands were determined and, finally, the concentrations of both O^2−^ and OH^−^ on the surfaces of ZrO_2_, CeO_2_ Al_2_O_3_, CuO, ZnO, Ga_2_O_3_ and MgO were determined. We also elaborated a new method for determination of the total concentration of all the basic sites (O^2−^ plus OH^−^) on oxides via the desorption of CO_2_ monitored by IR. The sum of concentrations of O^2−^ and OH^−^, determined separately in our IR experiments, was very close to total basic sites concentration determined in desorption studies [18,19].

The present study concerns the problem of modification of basic properties of alumina by the adsorption of ethanol and ammonia prior to adsorption of CO_2_. Our previous studies [20] evidenced that the reaction of alcohols with surface Al-OH groups produced alcoxy groups. It was interesting to know how the substitution of Al-OH by Al-O-C_2_H_5_ would modify the basicity of neighbouring surface sites. Another problem was the study of effect of adsorption of ammonia, which interacts with Lewis acid sites (being three-coordinated Al) on the basicity of neighbouring surface sites. In this study, the information on the basicity of surface sites was obtained by adsorption of CO_2_ as probe molecule. The concentration of OH^−^ was determined from the intensity of the δ_OH_ band (1230 cm^−1^), and the extinction coefficient of this band determined in our previous study. [18]. The total basicity (concentration of O^2−^ plus OH^−^) was determined in desorption studies monitored by IR [19]. The concentration of O^2−^ was the difference between the total basicity (O^2−^ plus OH^−^) and the concentration of OH^−^. 

## 2. Results and Discussion

### 2.1. Basic Sites on Al_2_O_3_–CO_2_ Adsorption

**Figure 1:**
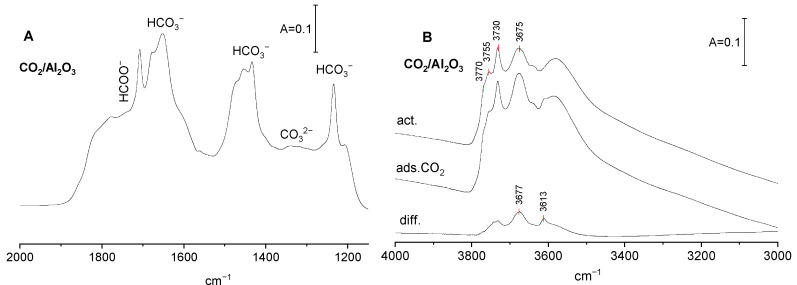
(**A**) The spectrum of CO_2_ adsorbed on Al_2_O_3._ (**B**) The spectra of OH groups on Al_2_O_3_ activated, upon CO_2_ adsorption and difference spectrum.

The spectrum of CO_2_ adsorbed on Al_2_O_3_ activated at 720 K is presented in Figure 1A. The band interpretation was based on the earlier results of Collins et al. [16]. The visualization of the vibration of carbonate species is given in [21]. The spectra show intensive bands of bicarbonate species: ν_sym C-O_ at 1435–1480 cm^−1^, ν_asym C-O_ at 1660–1680 cm^−1^, and the bending of OH group δ_OH_ at ca. 1230 cm^−1^. The band of ν_sym C-O_ is complex. It is composed of submaxima of monodentate and bidentate bicarbonates (1435 and 1455 cm^−1^ resp.). The high frequency component (at 1475 cm^−1^) cannot be clearly identified. It is not excluded that it can be assigned to ν_sym C-O_ of polydentate carbonate species. The bands of carboxylate species, ν_sym C-O_ at ca. 1200 cm^−1^ and ν_asym C-O_ 1700–1800 cm^−1^ [6], are present too. The bands of carbonate species (1300–1400 cm^−1^) are relatively weak. These results evidence that the basic sites on alumina surface are mostly basic OH^−^, the contribution of O^2−^ is rather low. Similar results were also obtained by other authors [22,23].The spectrum of OH groups of Al_2_O_3_ shows four distinct OH bands at 3770, 3755, 3730 and 3675 cm^−1^. The assignment of these hydroxyl bands was proposed by Knözinger and Ratnasamy [24]. The band at 3770 cm^−1^ was assigned to terminal Al-OH in which Al is four-coordinated, 3755 cm^−1^, to bibridged hydroxyls in which both Al are hexacoordinated, 3730 cm^−1^, to bibridged hydroxyls in which one Al is four-coordinated and the second one is hexacordinated. The 3675 cm^−1^ band was assigned to tribridged hydroxyls in which all three Al are hexacoordinated. The band at 3585 cm^−1^ may be due to oxohydroxy species. These free OH groups were denoted as: type I, IIB, IIA, and III.

According to Knozinger et al. [24], they differ in the electrical charge: those of type I are the most negative, and those of type III are positive. The reaction of OH groups with CO_2_ causes appearance of new C-OH bands typical of bicarbonate species. The band at 3613 cm^−1^ may be assigned to bidentate, and the band at 3677 cm^−1^ to monodentate bicarbonates. 

### 2.2. Basic Sites on Al_2_O_3_ with Preadsorbed Ethanol

The reaction of alcohols with surface hydroxyls on oxides produces alkoxyl groups according to the scheme: X-OH + HO-R = X-O-R + H_2_O. In order to study how the substitution of surface Al-OH by Al-O-C_2_H_5_ changes the basic properties of alumina, CO_2_ was adsorbed on alumina on which ethanol was preadsorbed at room temperature and, subsequently, non-reacting ethanol and water were removed by evacuation at 370 K.

**Figure 2:**
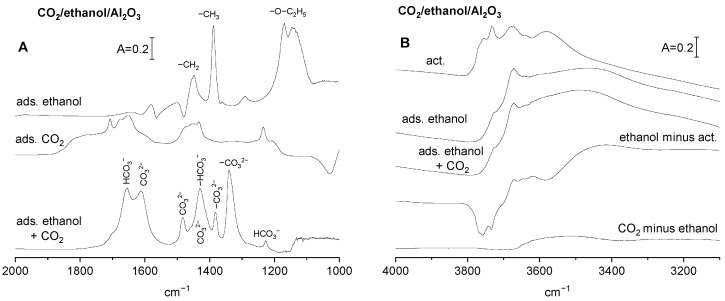
(**A**) The spectra of ethoxy groups recorded upon adsorption of ethanol and removal physisorbed molecules, the spectrum of CO_2_ adsorbed on activated Al_2_O_3_, and the spectrum recorded upon adsorption of CO_2_ on Al_2_O_3_ containing ethoxy groups. The spectrum of ethoxy groups has been subtracted. (**B**) The spectra of OH groups on Al_2_O_3_ activated upon the reaction with ethanol and upon adsorption of CO_2_ on Al_2_O_3_ containing ethoxy groups, as well as the difference spectra.

The spectra recorded upon the adsorption of ethanol on activated alumina followed by evacuation are presented in Figure 2A. The spectra show intense bands at 1130 and 1170 cm^−1^ of monodentate ethoxyls, as well as at the 1390 and 1450 cm^−1^ bands of the CH_3_ and CH_2_ groups. 

The spectra of hydroxyl groups are presented in Figure 2B. The formation of ethoxy groups is accompanied by the loss of some hydroxyls. The hydroxyls of highest stretching frequencies are the most reactive against ethanol. This agrees with the fact that, according to Knozinger et al. [24], high frequency hydroxyls are the most negative. 

The spectra of (bi)carbonate species formed by the reaction of CO_2_ with basic sites on alumina with preadsorbed ethanol (i.e., containing surface ethoxy groups) are presented in Figure 2A, and may be compared with the spectra of (bi)carbonates formed on alumina surface without ethoxyls. At the presence of ethoxy groups, the band of bending of OH (δ_OH_ at 1230 cm^−1^) is lower than that without ethoxyls, indicating that the contribution of bicarbonates is lower too. This may be related to the fact that the reaction with ethanol consumed some of the most basic Al-OH, therefore the amount of basic OH^−^ able to react with CO_2_ is smaller. The spectra of (bi)carbonate species formed on alumina with preadsorbed ethanol (Figure 2A) show some new bands that were absent without ethanol. A possible interpretation of these new bands will be now discussed. The new bands at 1340 and 1615 cm^−1^ may be attributed to ν_sym C-O_ and ν_asym C-O_ in bidendate carbonates, respectively. The band at 1385 cm^−1^ may be assigned to monodentate carbonates, and the bands at 1430 and 1480 cm^−1^ to polydentate carbonates. The presence of relatively strong bands of carbonate species formed at the presence of ethoxyl groups is evidence that some surface oxygens that were not enough basic to react with CO_2_ became basic if Al-OH were substituted by Al-O-C_2_H_5_. The reaction of CO_2_ with basic sites affects only a few of the hydroxyl groups on alumina with ethoxy groups (Figure 2B, bottom spectrum). It may be explained by taking into account that some hydroxyls were already consumed by the reaction with ethanol and, therefore, a relatively small amount of bicarbonates was formed by the reaction of OH^−^ with CO_2_. 

The information on the concentration of OH^−^ on Al_2_O_3_ without and with ethoxy groups was obtained from the intensity of the δ_OH_ band (at 1230 cm^−1^) and its extinction coefficient, as determined in our previous study [18]. These values are presented in Table 1. The information on the total concentration of all the basic sites was obtained in studies of the desorption of CO_2_ monitored by IR spectroscopy by using a method described in our previous paper [19]. These values are also presented in Table 1. Assuming that the total concentration of basic sites is the sum of concentrations of OH^−^ and O^2−^, the concentration of O^2−^ can be calculated by subtraction. The values of O^2−^ concentration are also given in Table 1. According to the data given in Table 1, Al_2_O_3_ without ethoxy groups contains mostly OH^−^, the contribution of O^2−^ is significantly lower, and the formation of ethoxy groups by the reaction with ethanol produces important amounts of new O^2−^ able to react with CO_2_. This statement agrees with the conclusion obtained by comparing the spectra of CO_2_ adsorbed on Al_2_O_3_ without and with ethoxy groups (Figure 2A). The spectra suggest the appearance of new mono-, bi-, and polydentate carbonates on Al_2_O_3_ treated with ethanol.

### 2.3. Basic Sites on Al_2_O_3_ with Preadsorbed Ammonia

**Figure 3:**
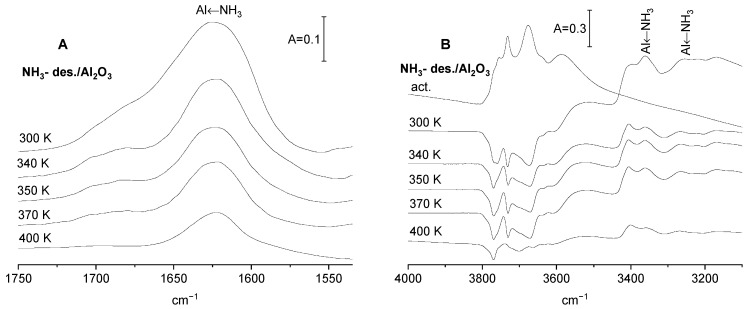
(**A**) The spectra of ammonia adsorbed on Al_2_O_3_ recorded upon the desorption at 300, 340, 350, 370 and 400 K. (**B**) The spectrum of OH groups on Al_2_O_3_ activated and difference spectra (spectra recorded upon the adsorption of ammonia and desorption at 300, 340, 350, 370 and 400 K minus spectrum of activated Al_2_O_3_).

The spectrum of ammonia adsorbed on alumina (Figure 3) shows several bands. The bands typical of ammonia interacting with Lewis acid sites are three-coordinated surface Al atoms as follows: 1620 cm^−1^ (antisymmetric deformation of NH_3_), 3270, and 3360 cm^−1^ (symmetric and antisymmetric N-H stretching). The presence of such three-coordinated Al atoms was evidenced by Knozinger and Ratnasamy [24]. These bands decrease as the ammonia is desorbed by evacuation at increasing temperatures (Figure 3). Ammonia adsorbed on alumina also interacts with Al-OH groups via hydrogen bonding. The minima on the difference spectra (Figure 3B) correspond to these Al-OH groups that are engaged in hydrogen bonding. There is no proton transfer from Al-OH to NH_3_, because the band of the ammonium ion (at ca. 1430 cm^−1^) is absent. Evacuation at increasing temperatures (Figure 3B), which removes ammonia from Al sites (decrease of 1620 cm^−1^), also removes ammonia from OH groups. The minima in the difference spectra in Figure 3B become more “shallow”. However, it should be noted that even the desorption at relatively high temperature (400 K) did not remove all the NH_3_ from OH groups (Figure 3B, bottom difference spectrum). The interaction of CO_2_ with Al_2_O_3_ with preadsorbed ammonia from which ammonia was partially removed by desorption at various temperature was followed.

**Figure 4:**
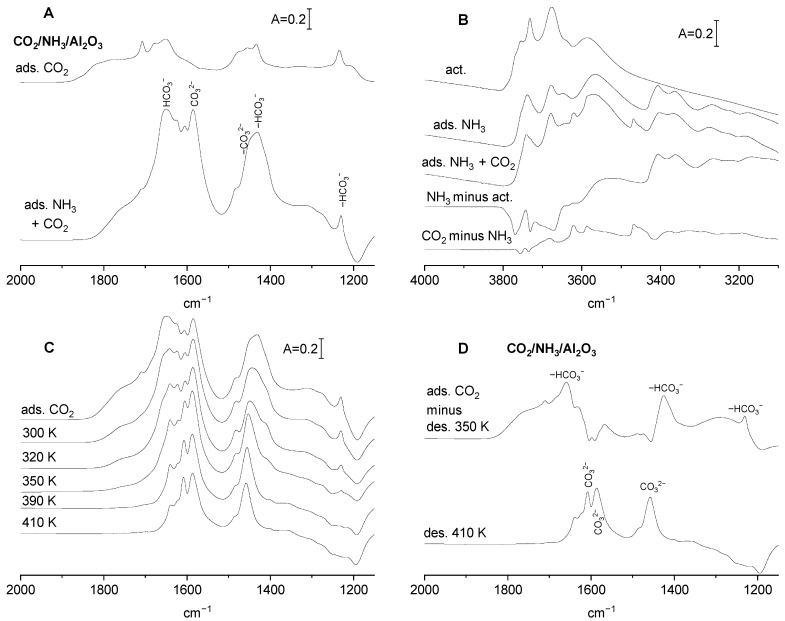
(**A**) The spectra of the adsorbed CO_2_ on activated Al_2_O_3_ and Al_2_O_3_, on which NH_3_ was adsorbed and, subsequently, desorbed at 340 K. (**B**) The spectrum of OH groups on Al_2_O_3_ activated, spectra recorded upon adsorption of NH_3_, followed by desorption at 340 K, adsorption of CO_2_ on Al_2_O_3_ with preadsorbed NH_3_, as well as difference spectra. (**C**) The spectra of (bi)carbonate species on Al_2_O_3_ with preadsorbed ammonia recorded upon the desorption at 300, 320, 350, 390 and 410 K. (**D**) (Bi)carbonates on Al_2_O_3_ with preadsorbed NH_3_. Spectrum of the less stale species (spectrum upon adsorption of CO_2_ minus spectrum upon desorption at 350 K). Spectrum of the most stable species (remaining upon the desorption at 410 K). Spectra are normalized to the same band intensity.

The spectra of CO_2_ adsorbed on alumina without and with preadsorbed ammonia are presented in Figure 4A. The excess ammonia (sufficient to saturate all the adsorption sites) was adsorbed at room temperature, and the physisorbed molecules were removed by evacuation at 340 K. CO_2_ was subsequently adsorbed on such a pretreated sample. The intensity of the δ_OH_ is comparable for alumina without and with ammonia; however, the intensity of carbonate bands is significantly higher in the presence of ammonia. It suggests that the neutralization of Lewis acid sites (three-coordinated Al) and engaging of some Al-OH in hydrogen bonding with ammonia increases the negative charge on neighbouring oxygens, which became sufficiently basic to react with CO_2_, forming CO_3_^2−^. The amount of OH^−^ reacting with CO_2_ does not change.

In order to get information on the assignment of IR bands of (bi)carbonate species formed on alumina at the presence of ammonia, the stability of these species was studied. Our previous study [18] evidenced that bicarbonates are less stable than carbonates, they decompose by evacuation at relatively low temperatures, and the IR bands that disappear via low temperature evacuation can, therefore, be assigned to bicarbonataes, and those that remain upon evacuation at higher temperatures can be assigned to carbonates. CO_2_ was adsorbed on alumina with preadsorbed ammonia and, subsequently, (bi)carbonates were decomposed by evacuation at 300, 320, 350, 390 and 410 K. The spectra recorded upon each desorption step are presented in Figure 4C, and the difference spectra in Figure 4D. The top spectrum in Figure 4D is the difference between the spectra recorded upon the adsorption of CO_2_ and evacuation at 350 K. This spectrum represents weakly bonded bicarbonate species; therefore, the bands at 1230, 1410 and 1660 cm^−1^ can be assigned to bicarbonates. The bands remaining upon evacuation at a relatively high temperature (410 K—bottom spectra in Figure 4D) at 1460, 1590 and 1610 cm^−1^ are due to polydentate carbonates. This interpretation is supported by the data presented in previous papers [11,15,16].

Summing up, it can be said that, in the presence of preadsorbed ammonia, new basic sites O^2−^ are formed, and they react with CO_2_, forming mostly polydentate carbonyls. 

The information on the concentration of basic sites OH^−^ and O^2−^ was obtained by taking into account the intensity of the δ_OH_ band (at 1230 cm^−1^) and its extinction coefficient, as well as the total concentration of basic sites, which was determined in desorption experiments.

More details are given in the previous chapter, and the concentration values are presented in Table 1. Similarly, as for ethanol, the preadsorption of ammonia produces a significant amount of new basic O^2−^. We suppose that this is the result of the transfer of electrons from basic ammonia molecule to Lewis acid sites (three-coordinated Al) next to neighbouring surface sites, which makes these neighbouring oxygen atoms sufficiently basic to react with CO_2_, forming CO_3_^2−^.

**Figure 5:**
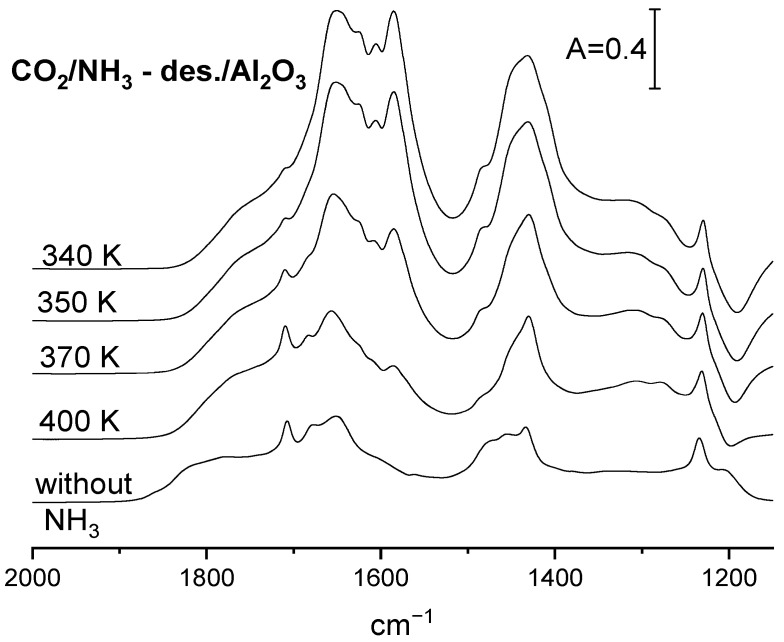
The spectra of (bi)carbonate species formed on Al_2_O_3_ with preadsorbed ammonia. The CO_2_ was adsorbed upon adsorption of NH_3_, followed by desorption at 340, 350, 370 and 400 K.

In order to study how the basicity of the alumina surface depends on the amount of ammonia adsorbed, all the acid sites were saturated by ammonia at room temperature, and ammonia was subsequently desorbed by evacuation at temperatures 300, 340, 350, 370 and 400 K. The spectra of adsorbed ammonia recorded upon each desorption step are presented in Figure 3, and the fraction of the Lewis acid sites still bonding ammonia upon such desorption calculated from the intensity of the 1620 cm^−1^ band is given in Table 2. CO_2_ was next adsorbed on such a pretreated sample, and the IR spectra are presented in Figure 5. The intensity of the carbonate bands decrease with the desorption temperature. The same conclusion was obtained by considering the results of desorption studies monitored by IR spectroscopy (Table 2). The total basicity (i.e., the amount of CO_2_ desorbed) decreases with the ammonia desorption temperature. Summing up, it can be stated that the more ammonia remains on the alumina surface, the higher the basicity of surface oxygens measured by CO_2_ adsorption is. In another words, the more Lewis acid sites neutralized by ammonia, the bigger the extend of electron transfer to surface sites is, and the more surface oxygen is sufficiently basic to react with CO_2_.

## 3. Materials and Methods

Al_2_O_3_ (ACS reagent, >99.6% purity) were purchased from Sigma-Aldrich (St. Louis, MO, USA), ethanol (Sigma Aldrich), ammonia and CO_2_ (Linde, purity 99.98) were used.

For IR studies, all oxides were pressed into thin wafers of ca. 100–250 mg. Prior to IR experiments, wafers were evacuated in situ in an IR cell at 720 K for 30 min. CO_2_, ethanol and ammonia were adsorbed at room temperature. For CO_2_ adsorption, the gas pressure in the IR cell was ca. 5 Torr. The band of molecular CO_2_ was present in the IR spectra, evidencing that all basic sites reacting with CO_2_ were saturated. For ethanol adsorption, the pressure in IR cell was ca. 3 Torr. Ethanol was contacted with alumina wafer for 3 min, and was subsequently desorbed via a 30 min evacuation at 370 K. In the experiments of ammonia adsorption, the pressure in the IR cell was 1–2 Torr. Ammonia was contacted with the alumina wafer for 3 min and, subsequently, it was desorbed by evacuation at various temperatures: 300, 340, 450, 370 and 400 K. The fraction of Lewis acid sites still covered by ammonia upon desorption was defined as the ratio of intensity of NH_3_ band at 1620 cm^−1^ in the spectrum recorded upon the desorption and the intensity of this band upon the desorption at 300 K. In all experiments, the CO_2_ was adsorbed at room temperature on the wafer of alumina with preadsorbed ethanol and ammonia.

The method of determination of total basicity (the concentration of O^2−^ plus OH^−^) was described in detail in our previous paper [19]; some information on this method will be presented below. All the basic sites on alumina without and with preadsorbed molecules were first saturated with CO_2_ at room temperature. Next, the gaseous CO_2_ was removed from the cell, and physisorbed molecules were removed by 1 min evacuation at room temperature. CO_2_ bonded to basic sites was subsequently desorbed at 470 K from the sample and trapped in a cold trap. The molecules trapped in the cold trap were next adsorbed on the wafer of zeolite NaY (which is a very efficient adsorbent). The amount of CO_2_ adsorbed on NaY was calculated from the intensity of the CO_2_ band at ca 2300 cm^−1^ and the extinction coefficient of this band. The total amount of CO_2_ adsorbed on basic sites was calculated by taking into account the amount of CO_2_ adsorbed on NaY, as well as the integrated intensities of all IR bands of the adsorbed (bi)carbonate species before and after evacuation at room temperature, and after the desorption at 470 K. More details are given in [19].

The spectra were recorded with a NICOLET 6700 spectrometer (Thermo Scientific, Cambridge, MA, USA) with a spectral resolution of 1 cm^−1^. The spectra were recorded at the transmission mode.

## 4. Conclusions

The experiments of CO_2_ adsorption evidenced that basic sites of OH^−^ were present on the alumina surface, and the concentration of basic O^2−^ sites was relatively low. The reaction of surface hydroxyls Al-OH with ethanol produced ethoxy groups Al-O-C_2_H_5_. At the presence of ethoxy groups, some surface oxygen become sufficiently basic to react with CO_2_, forming CO_3_^2−^. These new carbonates were mainly monodentate and bidentate. A similar situation was observed if NH_3_ was preadsorbed on alumina. Ammonia reacted with surface Lewis acid sites, i.e., three-coordinated Al atoms; the transfer of electrons from ammonia to Lewis acid site made neighbouring oxygens basic and able to react with CO_2_. The newly formed carbonates were mainly polydentate. Summing up, it can be stated that the basic properties of alumina (and, probably, also other oxides) can be modified by the coadsorption of some molecules. It is expected that it would also modify the catalytic properties of these materials.

## Figures and Tables

**Table 1:** The concentration of all the basic sites (total basicity OH^−^ + O^2−^), OH^−^, and of O^2−^ on Al_3_O_3_ without and with ethoxy groups, as well as with ammonia preadsorbed (physisorbed ammonia was removed by evacuation at 340 K).

	Concentration (µmol/g)		
	Total Basicity OH^−^ + O^2−^	OH^−^	O^2−^
Al_2_O_3_	45	38	7
Al_2_O_3_ + ethoxy gr.	60	18	42
Al_2_O_3_ + NH_3_	130	35	85

**Table 2:** The concentration of all the basic sites on Al_2_O_3_ with preadsorbed ammonia as a function of ammonia desorption temperature.

Desorption Temperature K	Fraction of Acid Sites Covered with NH_3_	Concentration of Basic Sites µmol/g
without NH_3_	0	45
340	0.68	110
350	0.52	95
370	0.41	71
400	0.24	62

## Data Availability

The data presented in this study are available in article.
